# Supplementary material for: Dual drug targeting to kill colon cancers
Source: Cancer Med. 2022 Mar 17;11(13):2612–26. doi: 10.1002/cam4.4641 (PMC9249985; doi:10.1002/cam4.4641)
Supplement: Supplementary file 1 — Appendix S1 [file CAM4-11-2612-s001.pdf]

Corona et al, Tables and Figures

Table 1: Effects of signaling inhibitors on proliferation and viability of three colorectal cancer cell lines in adherent cultures

	Proliferation Assay (IC_{50}) (µM)			Cytotoxicity Assay (EC_{50})* (µM)		
CRC Cell lines	SW620	LIM2537	LIM1899	SW620	LIM2537	LIM1899
Inhibitors:Target						
AG1478: EGFR	Inactive^{4}	8.9 ± 1.1^{3}	2 ± 0^{2}	-	>10	4.1 ± 3.2
WEHI-1208800:Src	0.1 ± 0.03^{3}	0.08 ± 0.03^{3}	0.1 ± 0.03^{3}	1.3 ± 0.9	0.23 ± 0.11	0.25 ± 0.05
LY294002:PI3K	>25^{2}	22.5±14^{3}	7.7±1.1^{3}	>40	>40	>40
ABT737:Bcl-2	15 ± 5^{3}	14.5 ± 3.5^{3}	10-20^{3}	10.6 ± 0.7	12.0 ± 3.6	11.6 ± 0.6
Pyrvinium pamoate:Wnt	5.2 ± 0.55^{3}	5.9 ± 1.34^{3}	4.9 ± 0.6^{2}	5.3 ± 1.7	6.3 ± 1.2	3.5 ± 1

* Superscripts indicate number of experiments; in each experiment there were 3 replicates

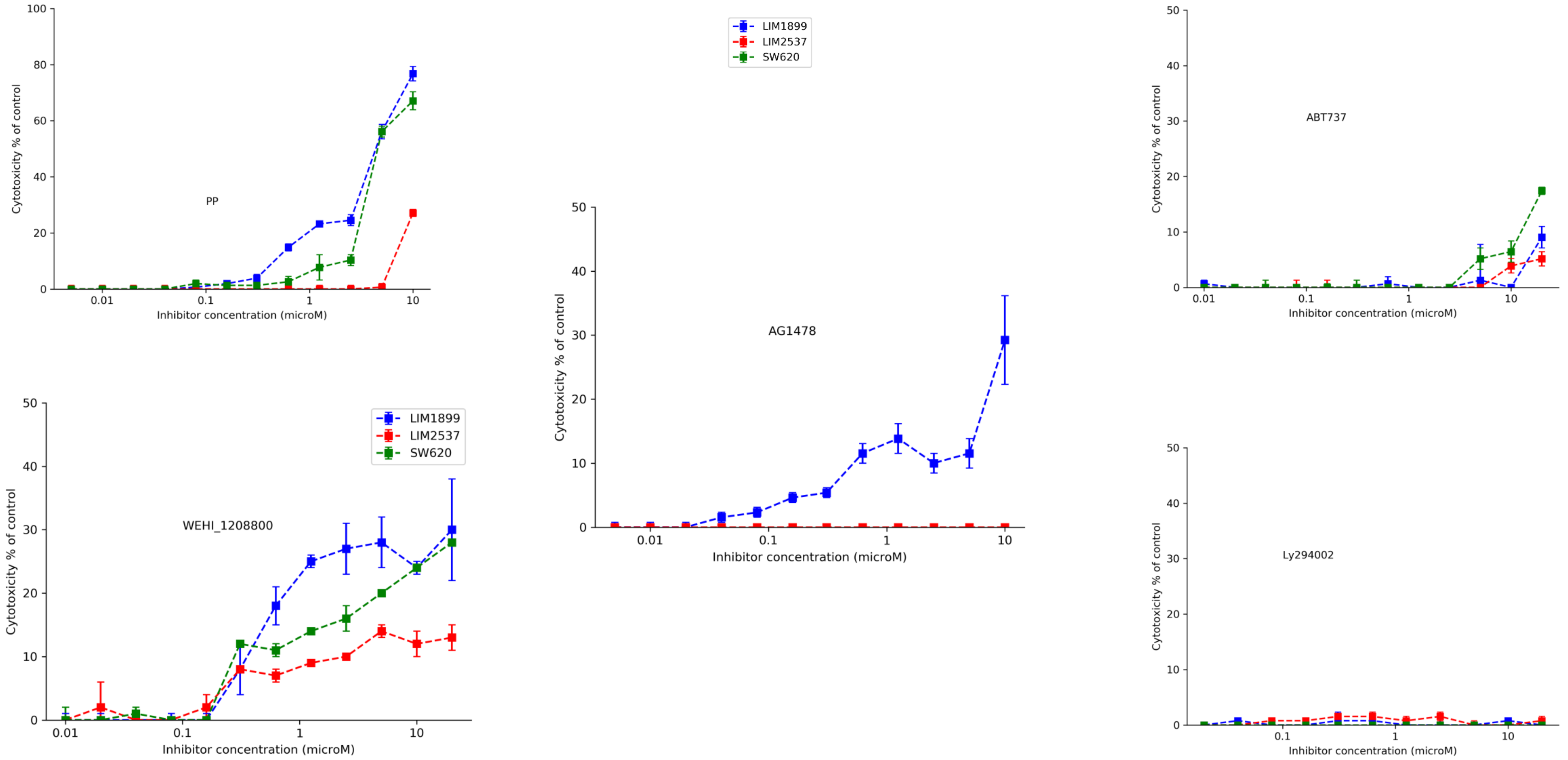

Figure 1 Adherent culture cytotoxicity of signaling inhibitors (pyrvinium pamoate PP, Wnt^{i}; AG1478, EGFR^{i},; WEHI-1208800,src^{i};ABT737;bcl2^{i}; and Ly294002,PI3K^{i}) on three CRC cell lines (LIM1899,LIM2537 and SW620)

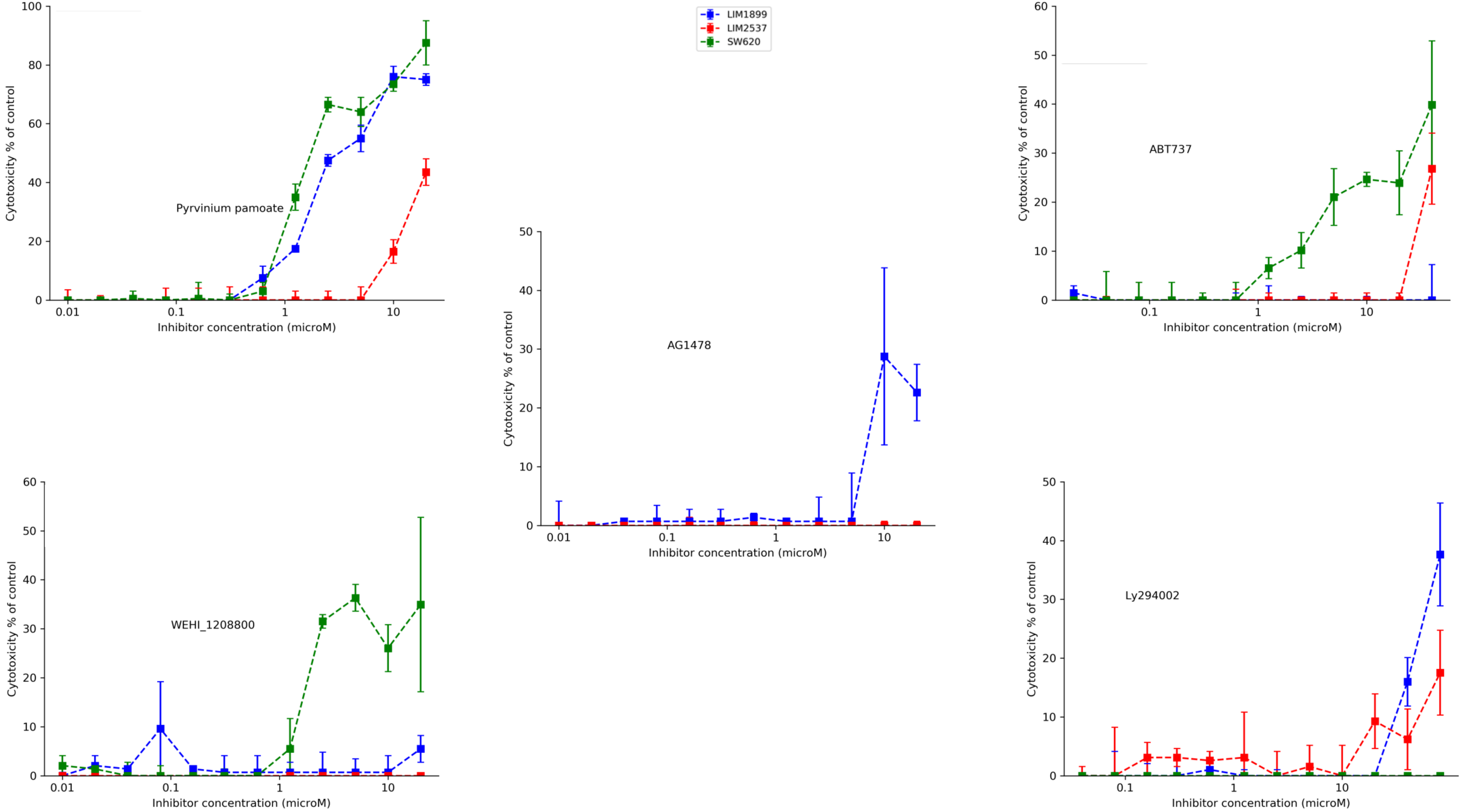

Figure 2 Non-Adherent culture cytotoxicity of signaling inhibitors (pyrvinium pamoate PP, Wnt^{i}; AG1478, EGFR^{i}; WEHI-1208800,Src^{i};ABT737;Bcl2^{i}; and Ly294002,PI3K^{i}) on three CRC cell lines (LIM1899,LIM2537 and SW620)

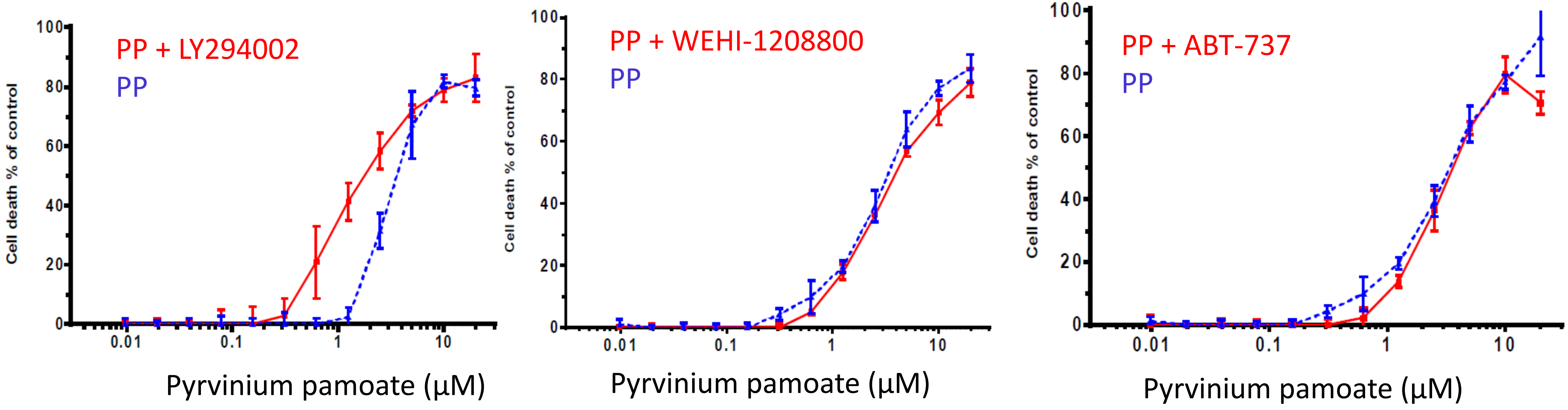

Figure 3 Cytotoxic effects of different concentrations of pyrvinium pamoate (PP) in combination with a fixed concentration of a PI3K inhibitor (LY294002,15μM) , a Src inhibitor (WEHI-1208800, 1μM) or the pro-apoptotic drug (ABT737, 10μM) on LIM1899 cells growing as colonospheres in hanging drop cultures.

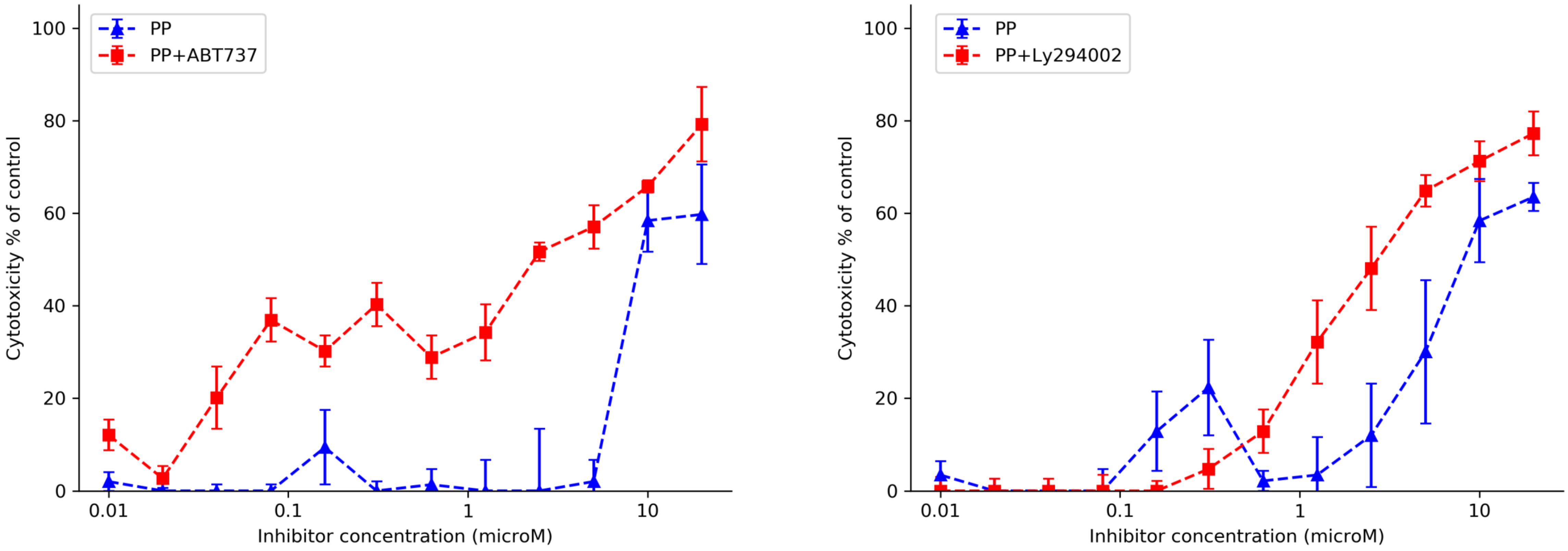

Figure 4 PI3K inhibitor (LY294002) and the pro-apoptotic drug (ABT737) increase the sensitivity of SW620 cells

to pyrvinium pamoate (PP)

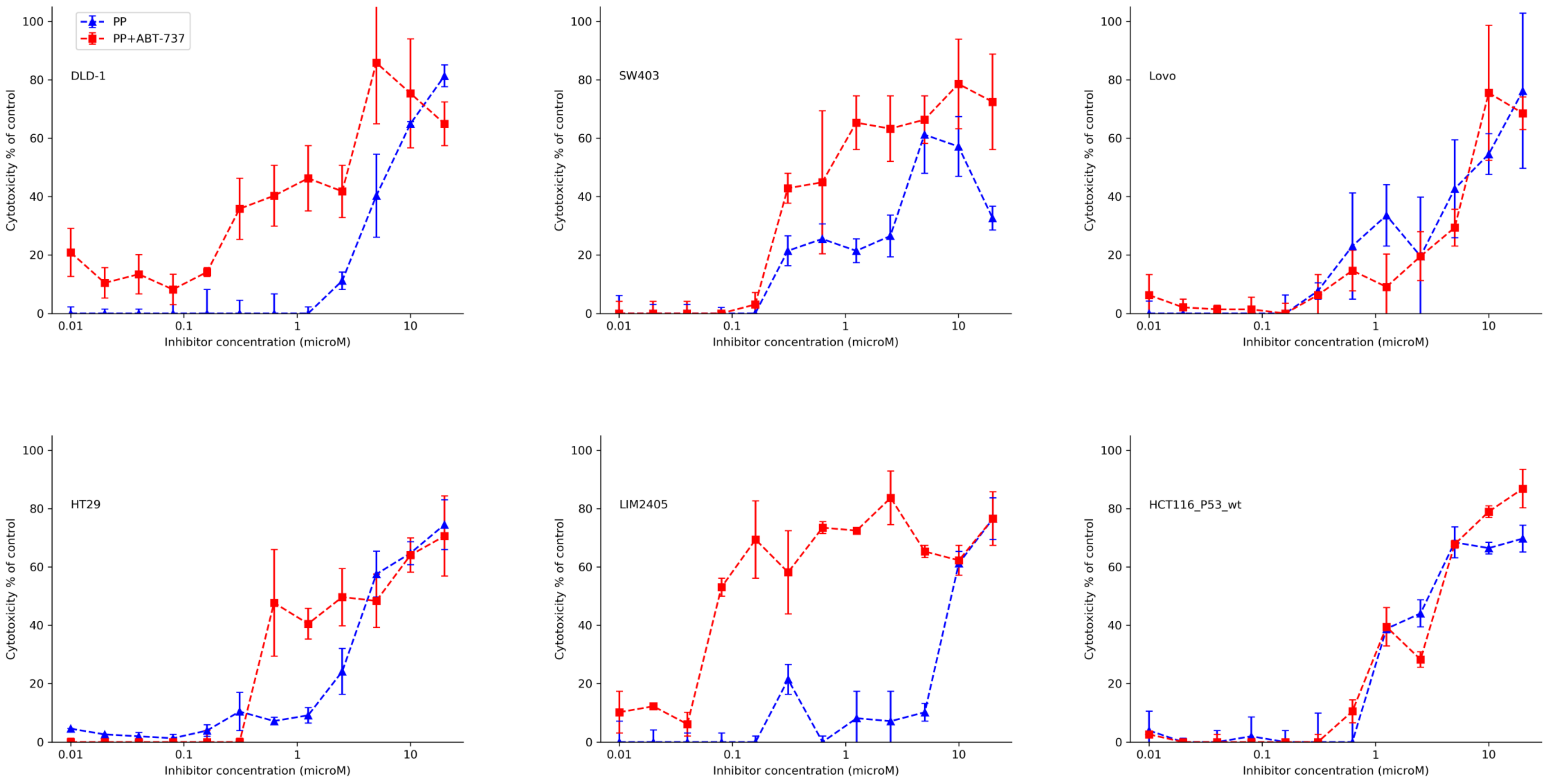

Figure 5 Cytotoxicity of pyrvinium pamoate (PP) in the presence or absence of ABT-737 (10μM) on six CRC cell lines

Table 2 EC_{50} for inhibitors as single agents and in combinations on CRC cell lines 3D cultures of colonospheres in hanging drops.

EC_{50} are the average of triplicates from 3 experiments ± SD

Inhibitors (μM) / Cell lines	PP	PP + WEHI-1208800	PP + LY294002	PP + ABT-737	WEHI-1208800	WEHI-1208800 + ABT-737	AG1478	AG1478 + ABT-737	LY294002	ABT-737
LIM1899	3 ± 0.1	2.7 ± 0.17	1.2 ± 0.06	2.6 ± 0.35			>20	>20	>30	>40
SW620	6.5 ± 0.5	4.3 ± 1.5	1 ± 0.85	1.2 ± 0.7	4.5 ± 3.5	5 ± 2.8			>40	6.6 ± 3.8

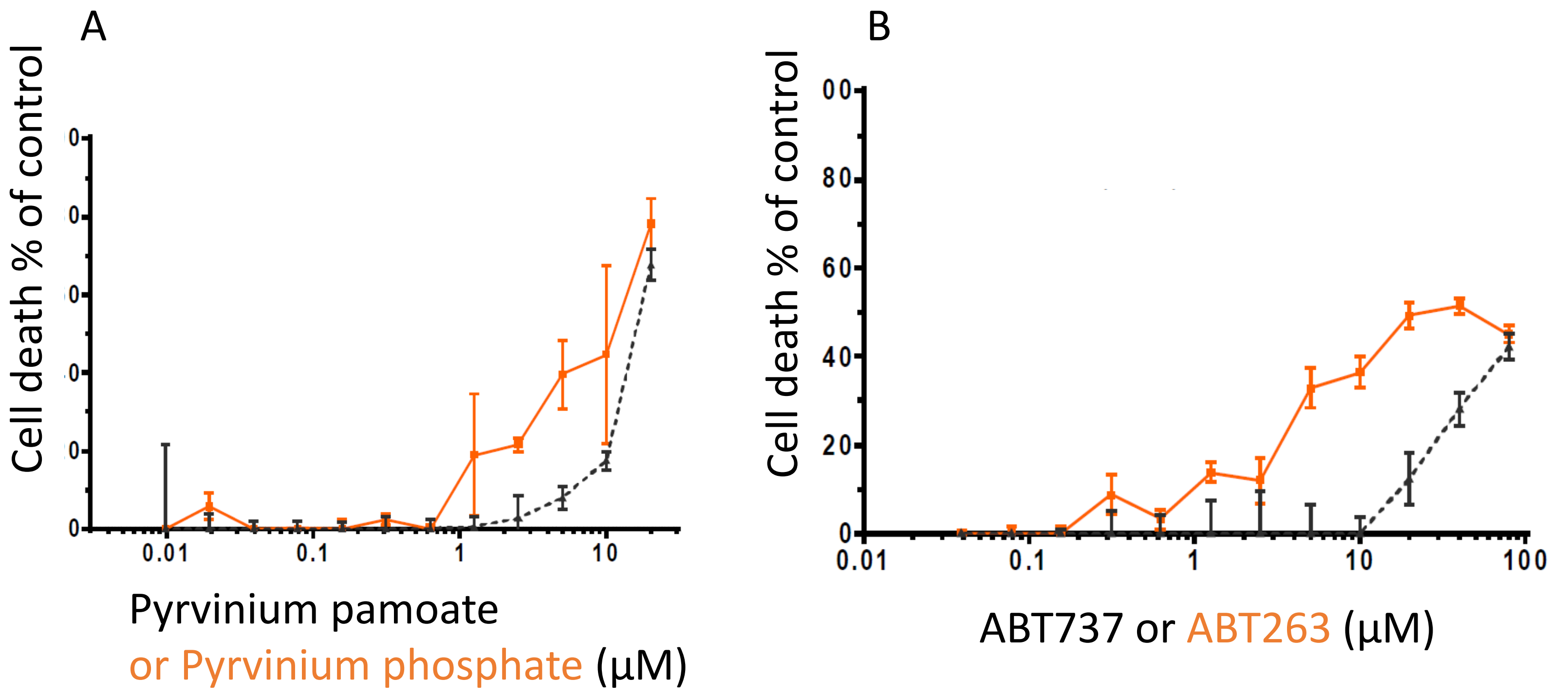

Figure 6 A, Effects of pyrvinium pamoate(PP) versus pyrvinium phosphate(PPh), B, ABT737 and ABT263 on the viability of the CRC cell line SW620 growing in 3D-hanging drop cultures,

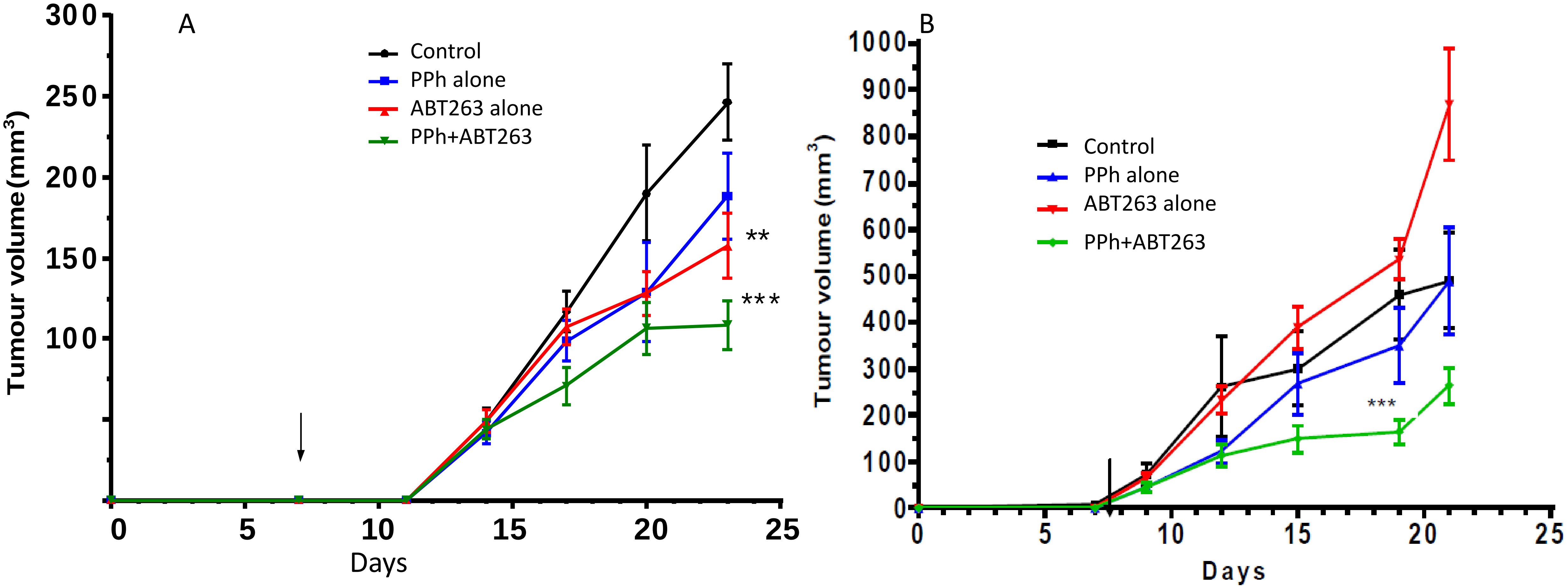

Figure 7 Effects of pyrvinium phosphate and ABT263 on the growth of SW620 tumor xenografts in A, female mice; B, male mice. The arrow shows the beginning of the therapy (day 7). The tumor volume was measured twice a week, with the mice under general sedation. Each point represents the average of the volume of all the tumors within a group. Error bars = S.E.M., *** = $p < 0.01$, ** = $p < 0.05$.

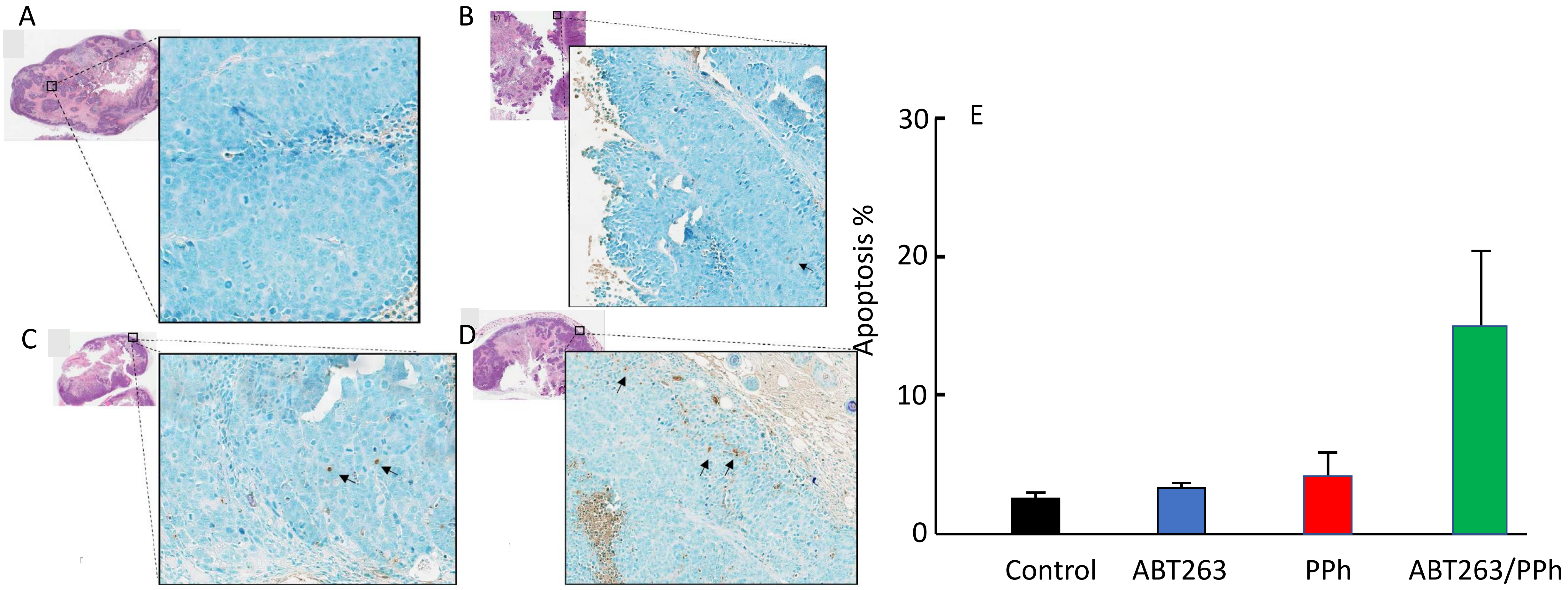

Figure 8 Detecting apoptotic cells (ApopTag IHC) in SW620 tumour xenografts treated with A, Vehicle, B, Pyrvinium phosphate (PPh), C, ABT263 and D, PPh +ABT263 The apoptotic cells stain brown; E, Percentage of apoptotic cells in SW620 tumors from the different treatments.

Supplementary Tables and Figures

Supplementary Table 1 Mutations in colorectal cell lines analyzed for drug sensitivity

Mutations Cell line	APC	β-catenin	K-Ras	B-Raf ex15	PI-3-K	p53
LIM2537	wt, stop1472	wt	wt	V600E	Δ exon13 (p85)	G245D
LIM1899	Wt	S45P	wt, G12A	wt	wt	wt
SW620	stop1338	wt	G12V	wt	wt	R273H + P309S

Supplementary Table 2 Summary of Signaling inhibitors used in this report to treat CRC cell lines

Drug	Mechanism of action	Bibliography
AG1478	EGFR inhibitor<br>Competitive inhibition of the ATP binding site of the kinase	Ellis *et al.*,<br>2006
WEHI 1208800	SRC Family Inhibitor<br>Competitive inhibition of the ATP binding site of the kinase	Patent No.<br>PCT/AU2011/000858,<br>8/07/2011
LY294002	PI3K inhibitor<br>Competitive inhibition of the ATP binding site of the kinase	Vlahos *et al.*,1994
ABT-737<br>ABT-263	Bcl-2 Family Inhibitor<br>Allosteric inhibition of the α-helix region (BH3)	Oltersdorf *et al.*, 2005
Pyrvinium Pamoate<br>Pyrvinium Phosphate	WNT Inhibitor<br>Activation of GSK-3β	Venerando et al., 2013
DAPT	NOTCH signaling inhibitor, γ-secretase inhibitor<br>Competitive inhibition of Preselenin	Morohashi *et al.*, 2006

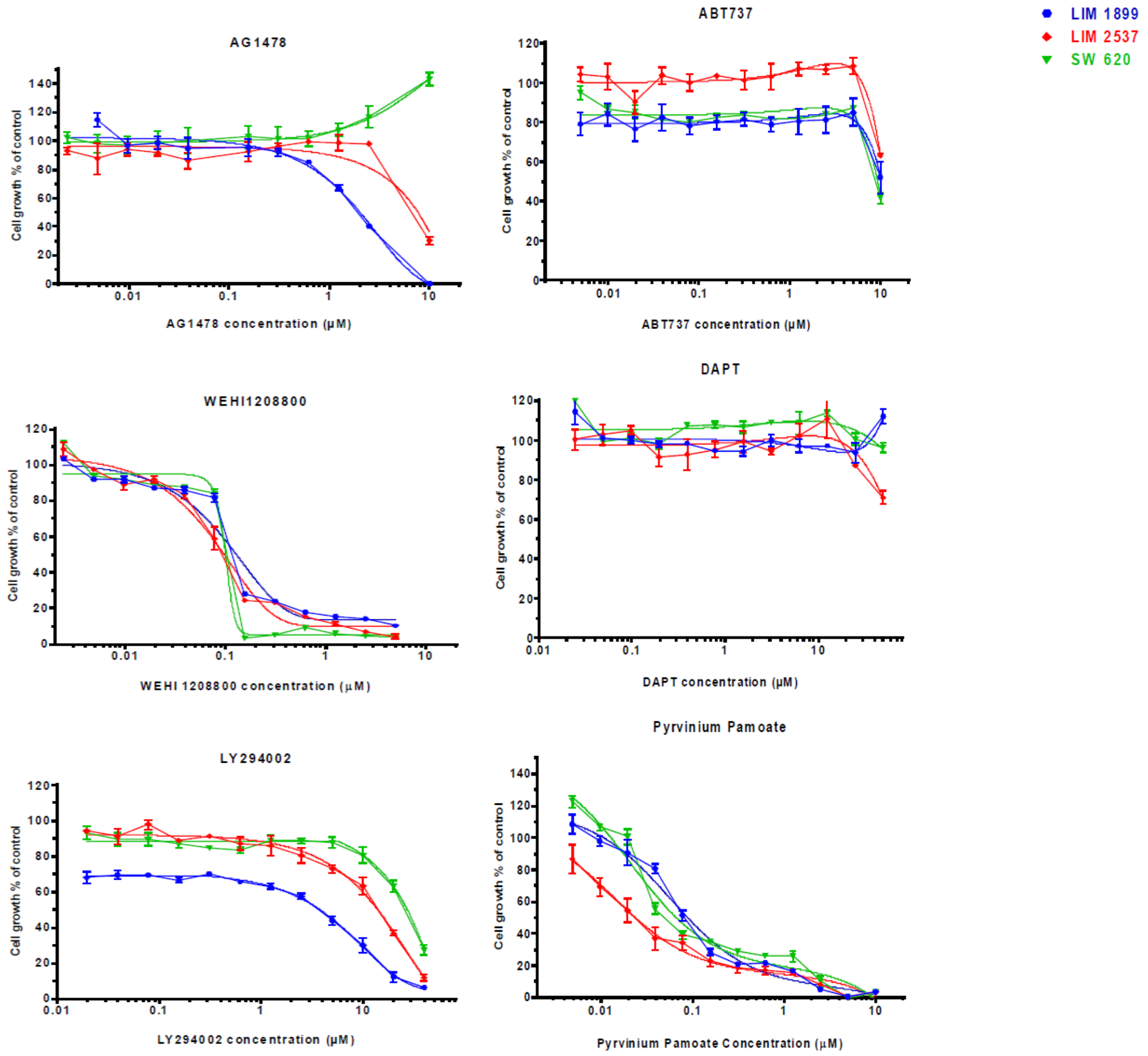

Supplementary Figure 1 The effects of different concentrations of signaling inhibitors (AG1478, EGFR[i]; ABT737, Bcl2[i]; WEHI1208800, Src[i]; DAPT, Notch[i]; LY294002, PI3K[i] and Pyrvinium pamoate, Wnt[i])on the proliferation of three CRC cell lines (LIM1899,LIM2537 and SW620) in adherent cell cultures, as measured using the MTT assay.

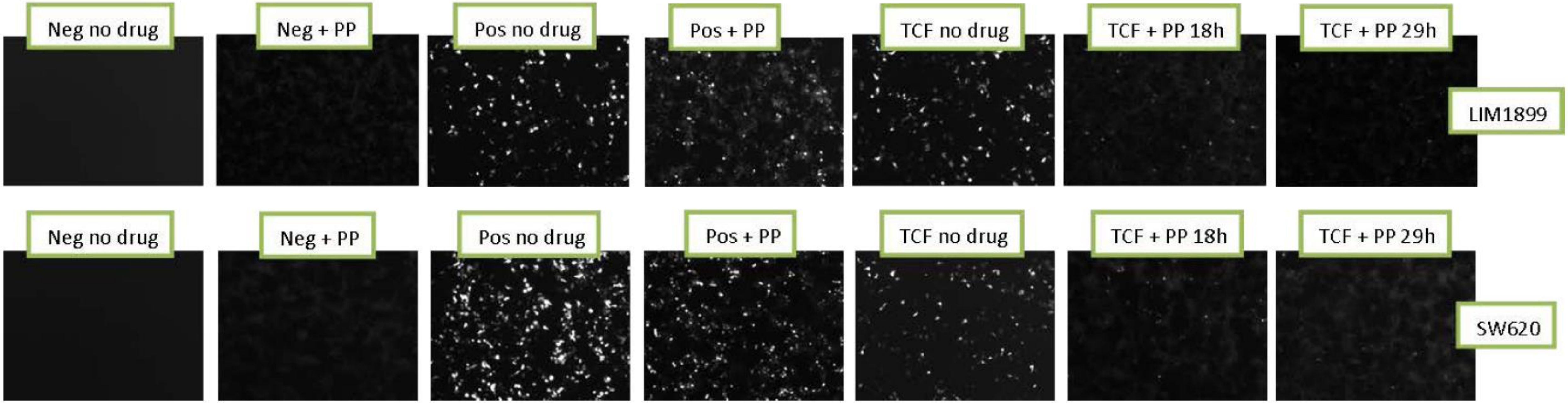

Supplemental Figure 2 : TCF-GFP Reporter assay indicates that pyrvinium pamoate inhibits Wnt signaling in LIM1899 and SW620 cells. SW 620 and LIM 1899 cells were plated at a density of 5 x 10^4 in 100µl of normal medium/well in 96 well/plates. Cells were transfected with 0.75µl of Fugene HD and 1µl of vector DNA (positive, negative or TCF-GFP) in 25µl of Optimem transfection medium. Both controls (positive and negative) were plated in duplicates, whereas the TCF-reporter was plated in triplicates. 24 hours after transfection PP was added to the cells at a concentration of 2µM. Cells were incubated for further 32h and imaged with a fluorescent microscope at two time-points (18h and 29h). The pictures were taken at the 18h time point unless otherwise specified. No fluorescence is evident in the negative controls, both with and without the drug. The number of fluorescent cells per field is not affected by the drug in the cells transfected with positive control. Cells transfected with the TCF-GFP vector show a consistent decrease in fluorescence when exposed to the drug for 18 and 29h. Images were taken using a Nikon C1 Confocal Microscope (10x) under fluorescent light.

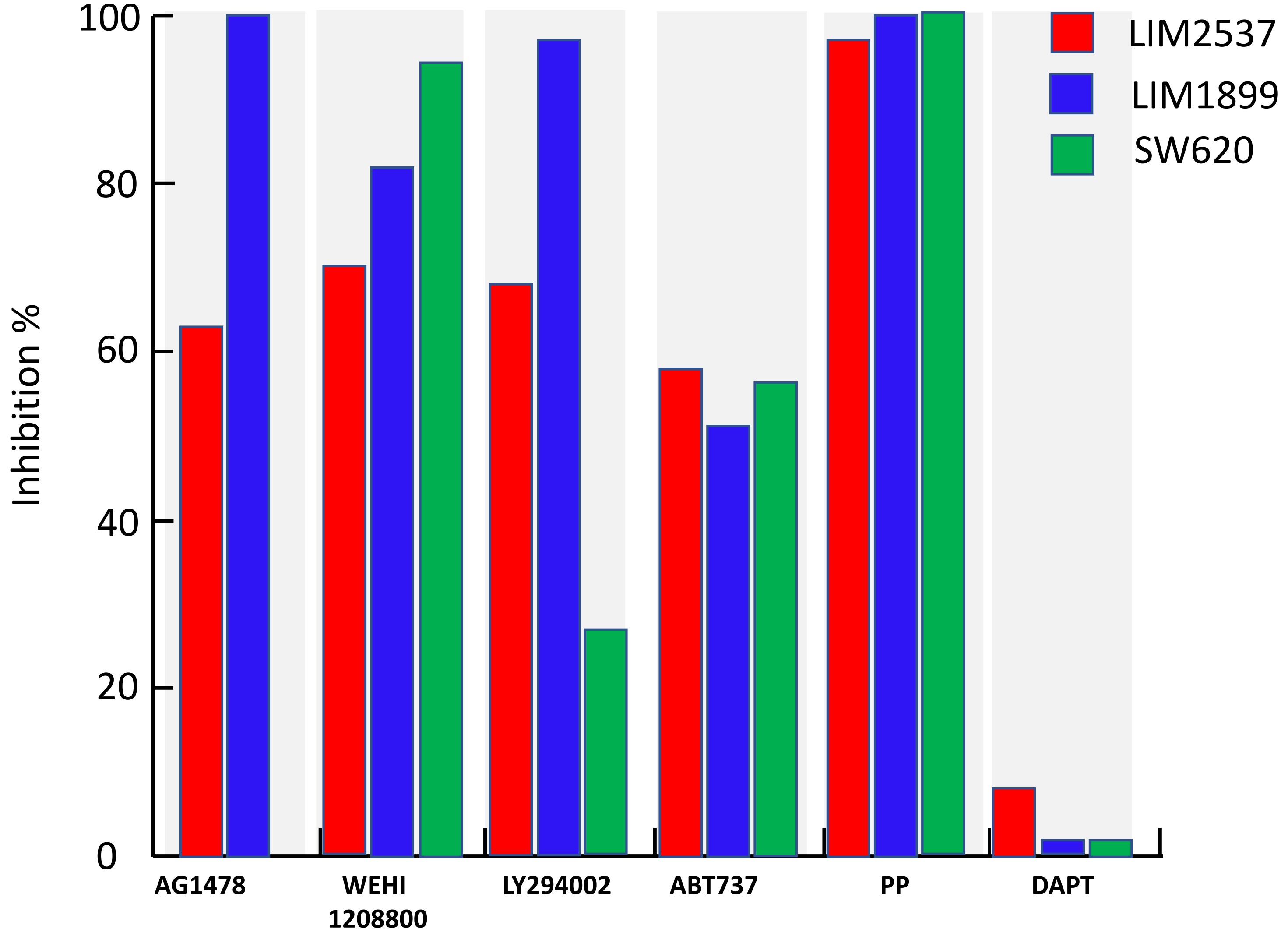

Supplementary Figure 3 The maximum proliferative inhibitions induced by each of the signaling inhibitors on three CRC cell lines in adherent cell assays (see details legend Supplementary Figure 1) are plotted as the mean of three experiments with triplicate assays for each experiment. Apart from the lack of effect of DAPT on the LIM1899 and SW620 cultures, all the results showed statistically significant inhibition in comparison to the control cultures (i.e. P<0.005).

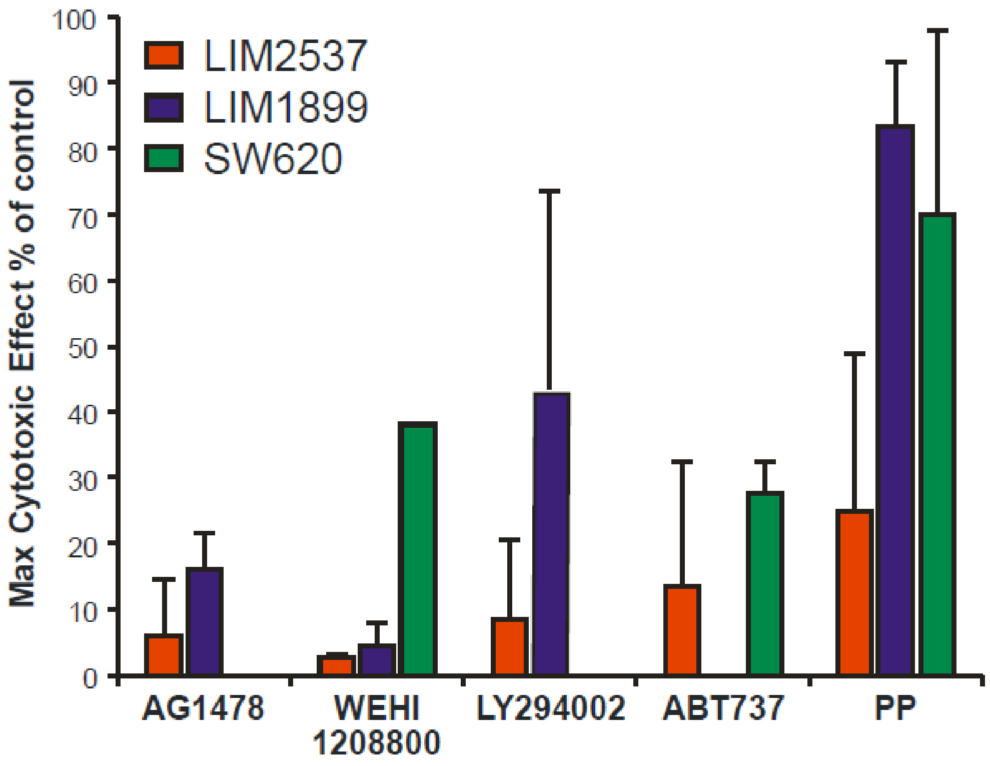

Supplementary Figure 4 Maximum cytotoxic effect of drugs on cells cultured as spheroids in Hanging Drops. Quantitation of the maximum percentage of apoptotic cell-death in comparison to controls, measured using the Cytotoxicity Detection Assay by Roche. Pyrvinium Pamoate induces 70% of cell death in SW 620 and LIM 1899 cell lines. All the other drugs are only moderately cytotoxic. LY294002 does not induce any cell death under these culture conditions. AG1478 is not cytotoxic on LIM 2537, whilst it was not tested with this assay on SW 620 because of the absence of EGFR on these cells. The graph shows the average results from 3 experiments, each experiment performed with triplicate wells. Data are drawn as mean ± SEM.

Supplementary Table 3: EC50s for cytotoxicity of the compounds for each cell line in cells grown in 3-D culture. Each experiment was done in triplicate, mean ± SD is shown.
WEHI = WEHI 1208800; PP = Pyrvinium Pamoate

Inhibitors (µM) EC_{50}	AG1478	WEHI	LY294002	ABT737	PP
LIM2537	6 ± 8.4	0.6 ± 0.1	23 ± 32.5	11 ± 15	14 ± 5.6
LIM1899	11 ± 3	8.6 ± 8	35 ± 18	>40	1.6 ± 0.9
SW620		0.9 ± 0.8	>80	6.6 ± 3.8	6 ± 2.3

Supplementary Table 4 Summary of inhibitor combinations tested on the three CRC cell lines: SW620, LIM2537 and LIM1899

Cell lines	Small Molecule Inhibitor Combinations
SW 620	PP + WEHI1208800<br>PP + ABT 737<br>PP + LY294002<br><br>WEHI1208800 + ABT 737
LIM 1899	PP + WEHI1208800<br>PP + ABT 737<br>PP + LY294002<br><br>AG 1478 + ABT 737
LIM 2537	WEHI1208800 + ABT 737

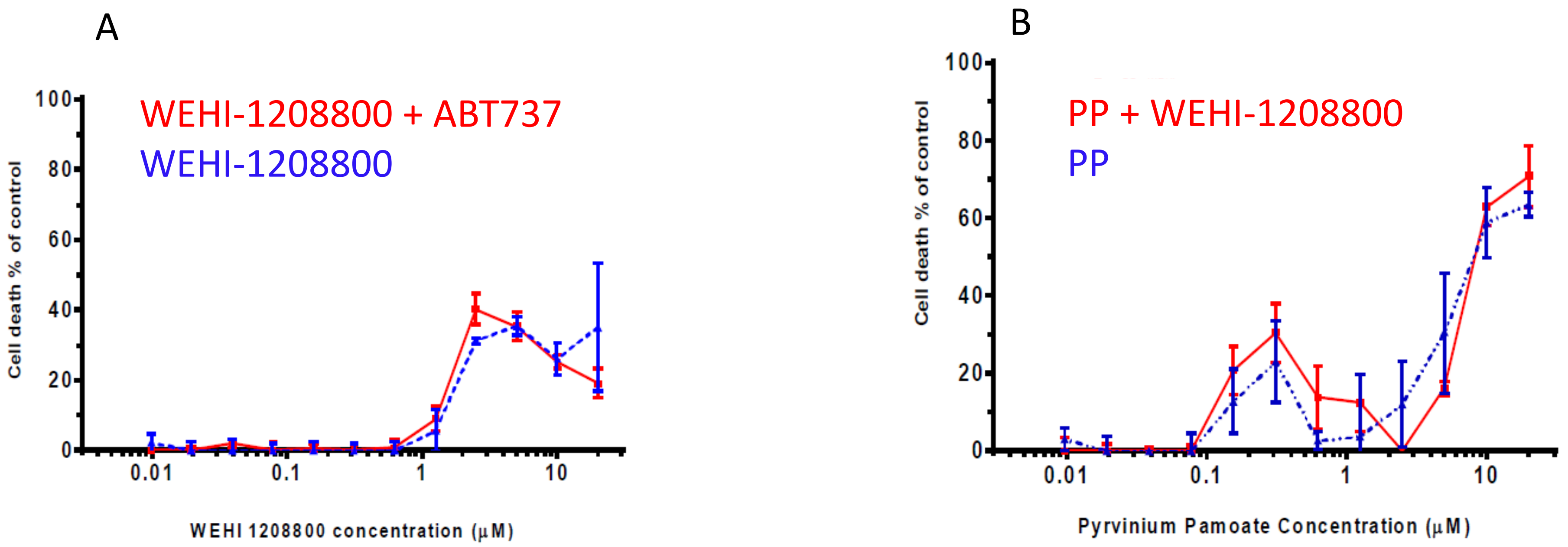

Supplementary Figure 5 A, WEHI1208800 and ABT737 (10µM) do not synergize when used in combination on SW620 cells; B, Pyrvinium pamoate and WEHI1208800 (1µm) do not synergize when used in combination on SW620 cells

Supplementary Table 5 Summary of Oncogene and Tumour Suppressor Gene profiles for CRC cell lines used in this study

Mutations / Cell line	APC	B-Catenin	K-Ras	B-Raf	PI3K	P53
DLD-1	*mut*	*wt*	*mut*	*wt*	*mut*	*mut*
LOVO	*mut*	*wt*	*mut*	*wt*	*wt*	*wt*
HT-29	*mut*	*wt*	*wt*	*mut*	*mut*	*mut*
LIM2405	*mut*	*wt*	*wt*	*mut*	*wt*	*wt*
SW403	*mut*	*wt*	*mut*	*wt*	*wt*	*wt*
HCT116	*wt*	*mut*	*mut*	*wt*	*mut*	*wt**

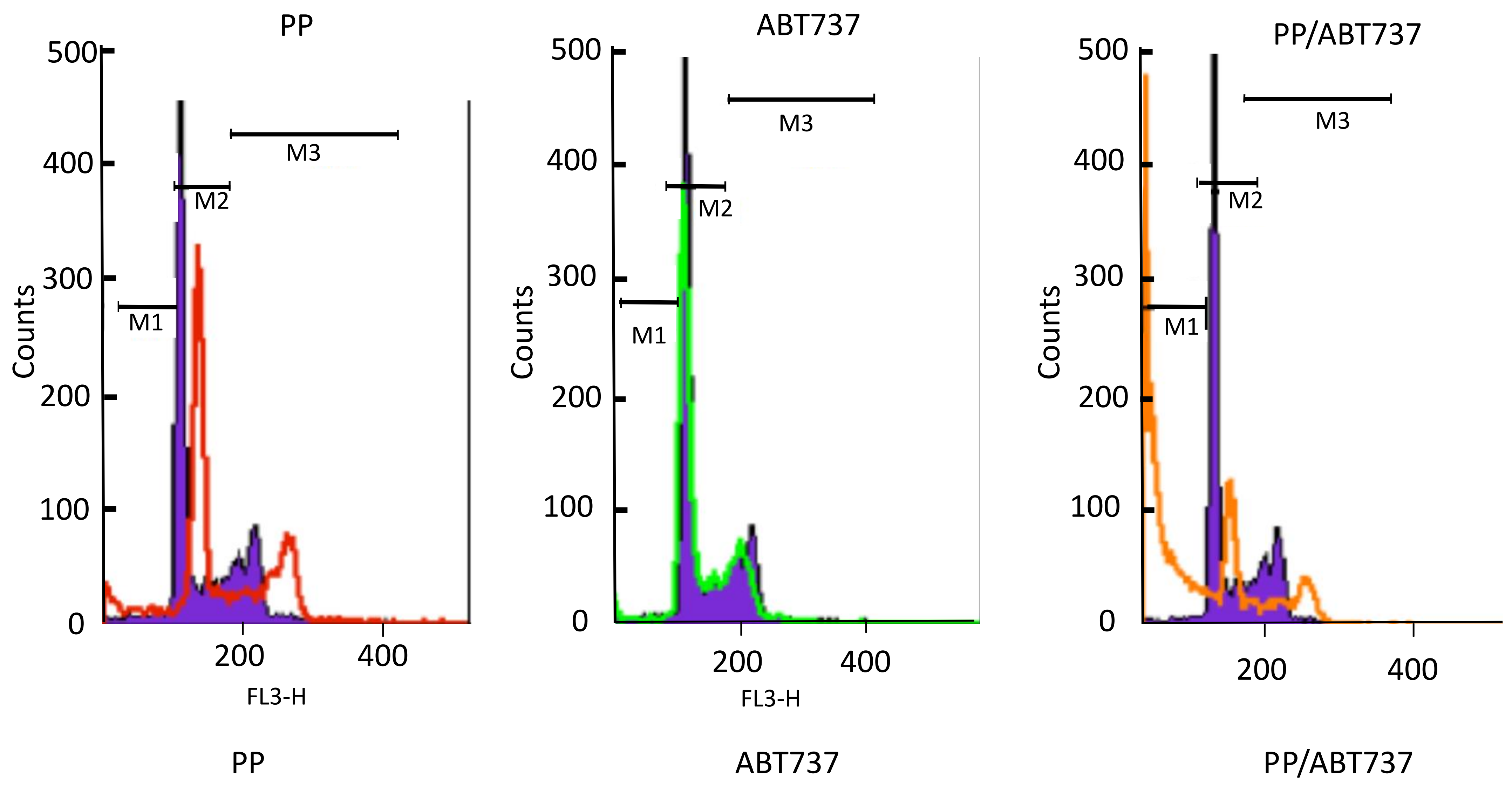

Supplementary Figure 6 Treatment of SW620 cells with PP/ABT737 induces cell death via apoptosis

A

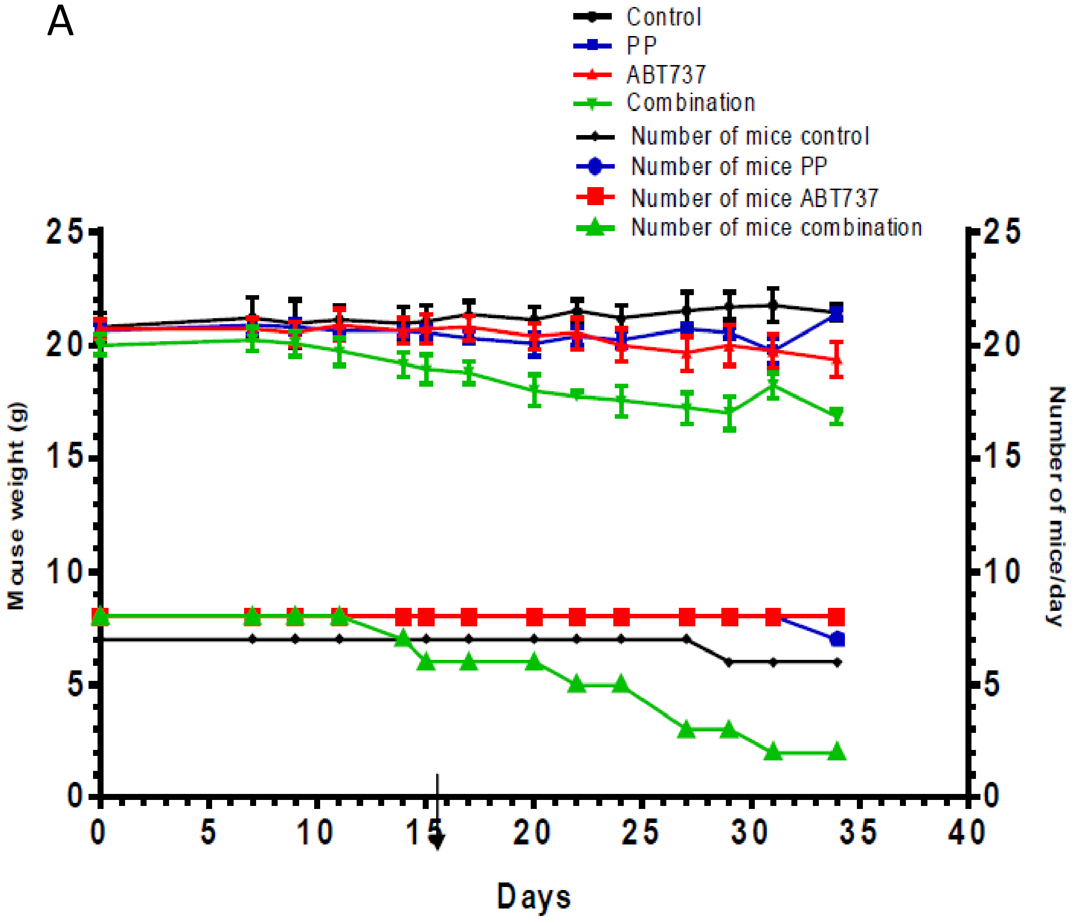

ight.

B

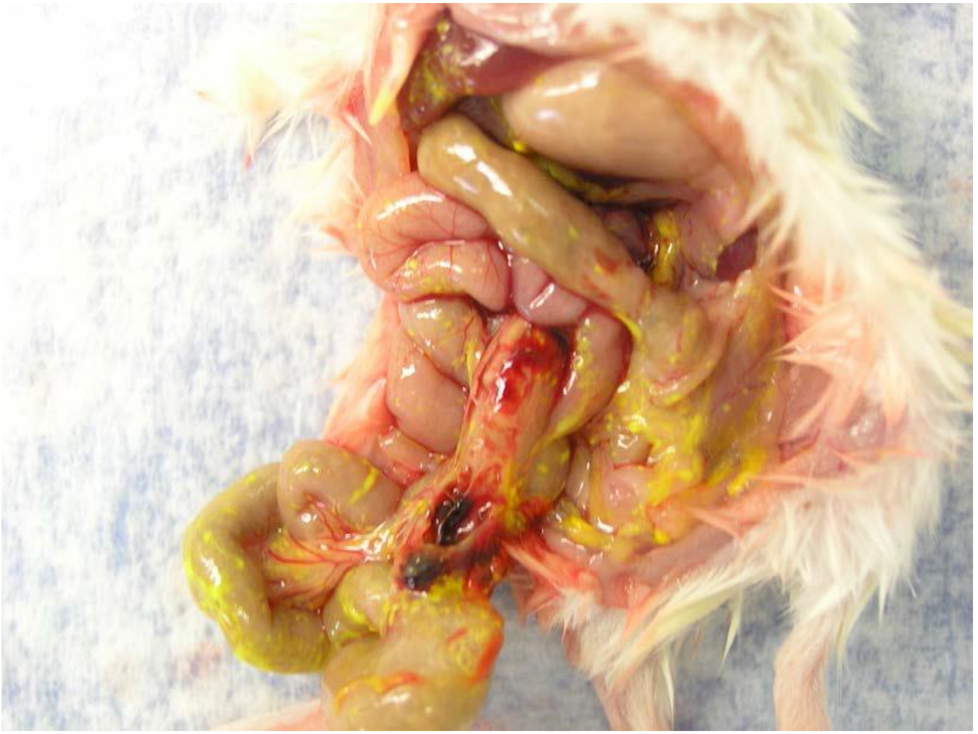

Supplementary Figure 7: A, Treatment of mice with the pyrvinium pamoate and ABT737 combination caused weight loss in mice; B, ABT737 injected intraperitoneally precipitated in the peritoneum (yellow precipitate).

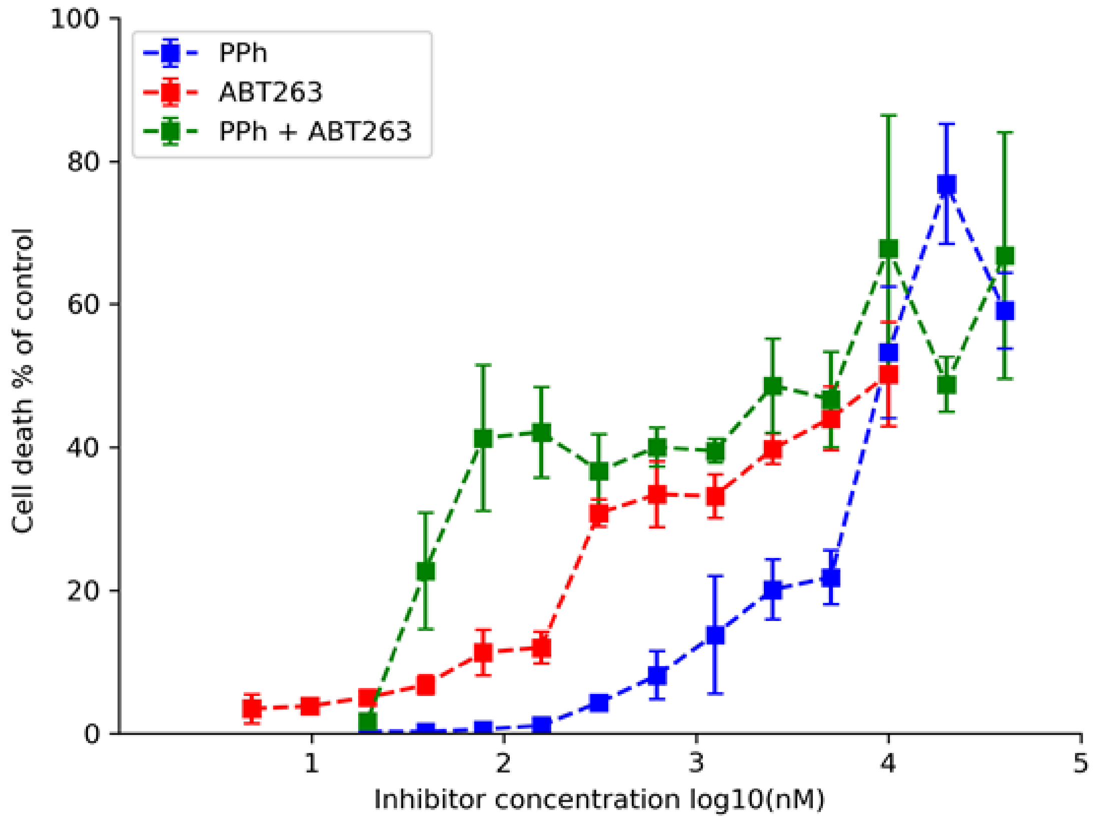

Supplementary Figure 8: Cytotoxicty effects of treatment with different concentrations of purvinium phosphate (PPh), ABT263 and the PPh/ABT263 (0.3um) combination on the CRC cell line LIM2405 growing in 3D-hanging drop cultures.

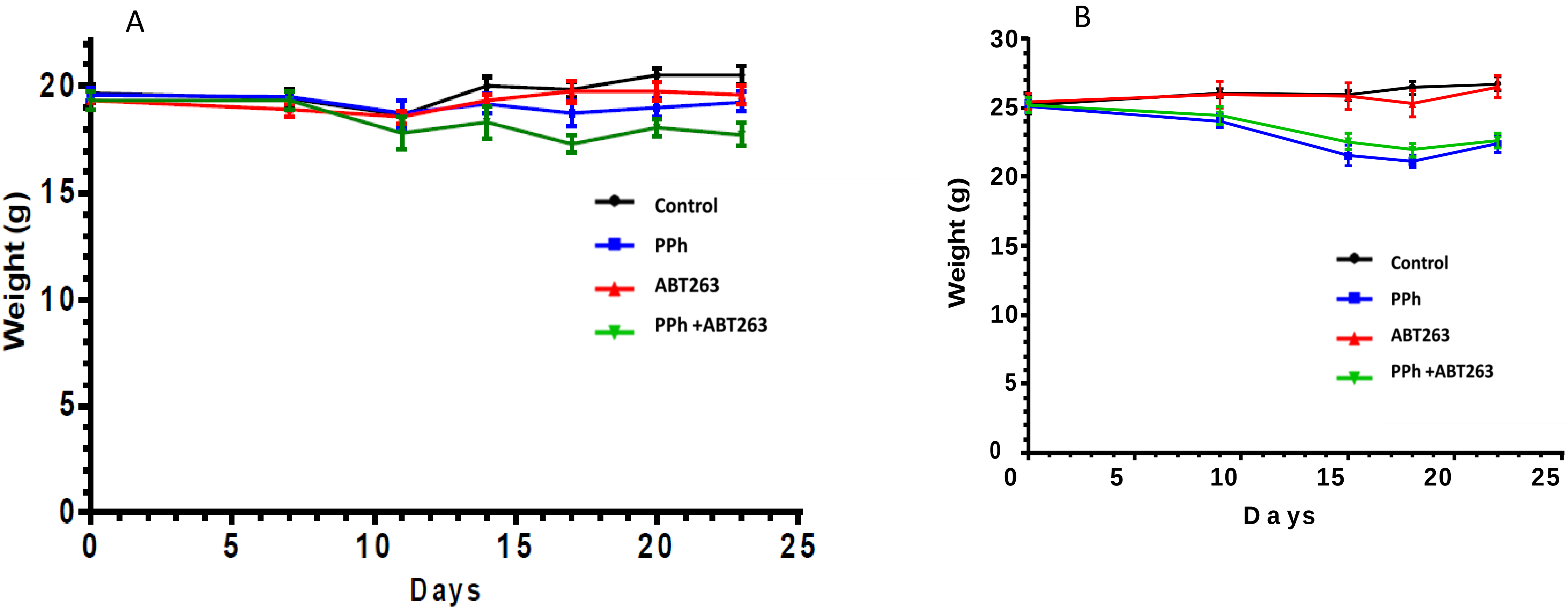

Supplementary Figure 9 A, Effect on Body Weight of treating **female** mice carrying SW620 xenografts with Pyrvinium phosphate (PPh) and ABT263. The mice were weighed twice each week. The combination group appeared to lose weight from day 8 to 10. The food for all of the mice was then supplemented with Sustagen; B Effect on Body Weight of treating **male** mice carrying SW620 xenografts with Pyrvinium phosphate (PPh)and ABT263. The mice were weighed twice a week. Two groups (PP and combination) appeared to lose weight from day 15 The food for all of the mice was then supplemented with Sustagen.
